# Non-Standard Risk Donors and Risk of Donor-Derived Infections: From Evaluation to Therapeutic Management

**DOI:** 10.3389/ti.2024.12803

**Published:** 2024-10-02

**Authors:** Paolo A. Grossi, Cameron Wolfe, Maddalena Peghin

**Affiliations:** ^1^ Infectious and Tropical Diseases Unit, Department of Medicine and Surgery, University of Insubria, Varese, Italy; ^2^ Division of Infectious Diseases, Duke University School of Medicine, Durham, NC, United States

**Keywords:** donor derived infections, emerging pathogens, HIV, hepatitis, SARS-CoV-2, bacteremia, multidrug resistant

## Abstract

Expected and unexpected donor-derived infections are a rare complication of solid organ transplantation, but can result in significant morbidity and mortality. Over the last years, the growing gap existing between patients on the waiting list and available organs has favored the use of organs from donors with suspected or confirmed infections, thanks to the improvement of risk mitigation strategies against transmission of well recognized and emerging infections. Given the recent developments, the particular interest of this review is to summarize data on how to maximize utilization of HIV+ donors in HIV+ recipients, the use of HCV-viremic donors and HBV positive donors. This article also covers the implications for recipient of organs from donors with bacteremia and the challenge of multidrug resistant (MDR) infections. Lastly this review describes emerging risks associated with recent Coronavirus Disease-2019 (COVID-19) pandemics.

## Introduction

Expected and unexpected donor-derived infections (DDI) remain an inherent risk of solid organ transplant (SOT) and are associated with significant morbidity and mortality, especially in the setting of parasitic and fungal diseases [1, 2].

The mitigation risk process for DDIs is based on the prevention of the transmission of infections with SOT with adequate safety simultaneously decreasing organ discard [3]. This complex methodological approach needs to adapt continuously to the changing landscape of infectious disease and the emerging evidence of new therapeutic and preventive options [4, 5]. While it is not an exhaustive list of potential pathogens impacting donors, the conditions demonstrate different approach to donor-derived disease mitigation. The aim of this review is to provide an update on DDIs to maximize organ utilization.

## Hepatitis B Positive Donors

The availability of effective antivirals with low risk of developing drug resistance and hepatitis B (HBV) vaccination have changed the epidemiology of HBV. All organ transplant candidates who are nonimmune to the virus, based on serologic testing, should be vaccinated against HBV infection. Active immunization against HBV in transplant candidates should be strongly encouraged not only because of the expected acquisition of protection against HBV, but also because it might allow the use of organs from HBV-positive donors [6]. Reducing the incidence of the disease has significantly reduced the carrier rates, HBV-related mortality (mainly due to cirrhosis and hepatocellular carcinoma) and the need for liver transplantation, allowing to expand the donor pool without impairing transplant outcomes [7–9].

**FIGURE 1:**
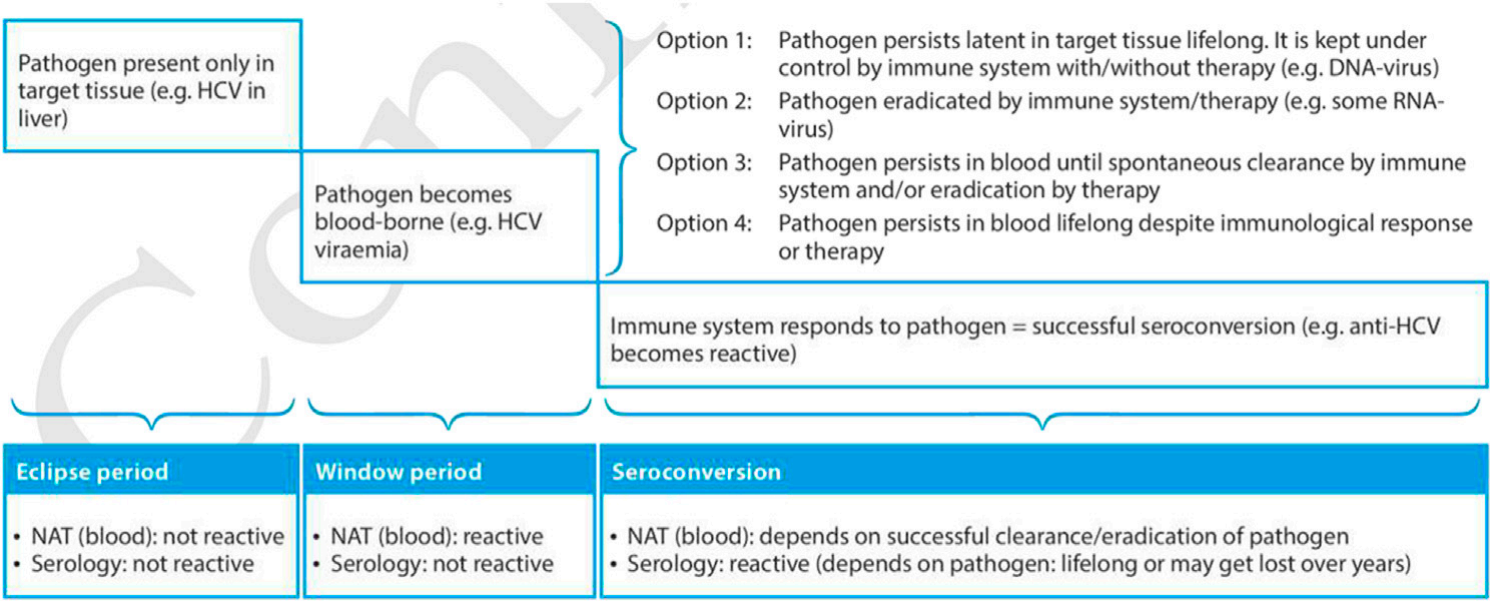
Timeline from infection until final seroconversion, including the eclipse period and window period. (Reproduced from EDQM Guide on Quality and Safety of Organs for Transplantation 8th edition, [11]).

Organ donors should be screened for serological evidence of HBV infection with chemiluminescence immunoassay (CLIA) techniques for HBV surface antigen (HBsAg) and core antigen antibodies (anti-HBc). In addition, nonstandard risk donors and donors with positive screening (HBsAg+ or anti-HBc+) should be screened for HBV infection by nucleic acid testing (NAT) (Table 1) (Figure 1) [10–12]. Of note that HBV antibody screening assays may not be reactive during the serologic window period (≈44 days), and NAT may also fail to detect the pathogen in the blood or plasma during the eclipse phase (≈20–22 days for HBV) [13].

**TABLE 1:** Behaviors at high risk of acquiring blood-born infections if present in the 30 days before organ procurement (10‐11‐12).

Non-standard risk donors
• Use of parenteral or inhaled drugs for non-medical reasons • Exposure to blood from a person suspected of being infected with HIV either by inoculation or by contamination of skin or mucous wounds • Incarceration (confinement in jail, prison, or juvenile correction facility) for ≥72 consecutive hours • Infants breastfed by an HIV‐infected mother • Children born from mothers infected with HIV, HBV or HCV • Unknown medical or social history • Sexual habits that can increase the risk of transmission of diseases o sexual relations with people affected or suspected of being affected by HIV, HCV, HBV o habitual and repeated sexual behavior (promiscuousness, casualness, sexual relations with the exchange of money or drugs) o sexual relations with people with a history of mercenary sex o sexual relations with subjects who have used parenteral or inhaled drugs o sex in exchange for money or drugs o people who have been diagnosed or have been treated for syphilis, gonorrhea, *chlamydia* or genital ulcers

All cases with potential risk of HBV transmission should be discussed with an expert [7, 14]. The most robust evidence on the risk of potential HBV transmission and related outcomes are with liver and kidney transplant, with very limited experience with thoracic transplant [7–9, 15, 16]. There is a lack of standardized antiviral prophylaxis and long term follow up.

**FIGURE 2:**
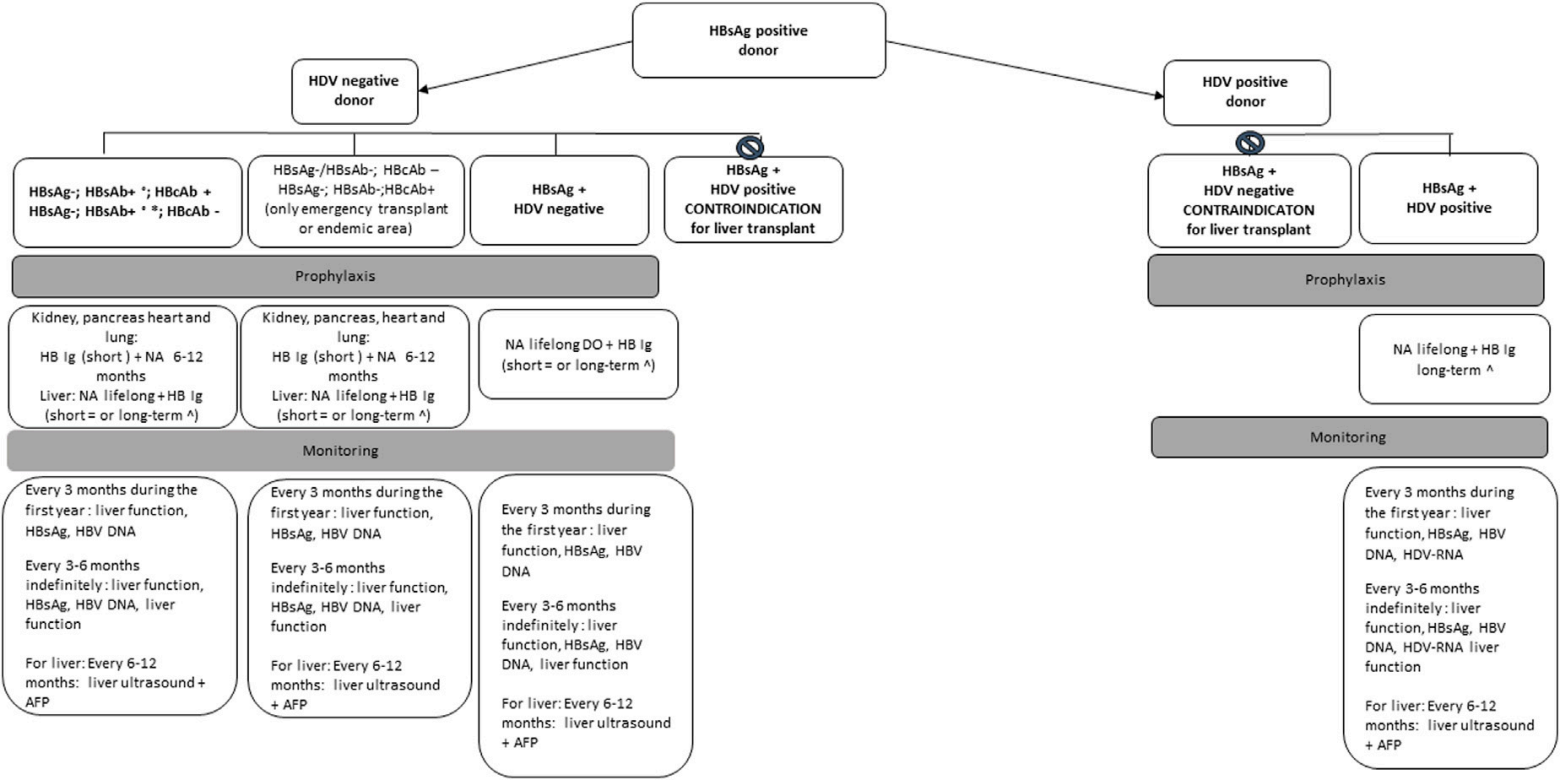
Management of recipients of organs from HBs Ag positive donor based on expert opinion recommendations.

The risk of transmission is well documented in donors with positive HBsAg (range 0.5%–7%) [17]. Transplantation from an HBsAg+ donor can be performed to an HBsAg+ recipient or with reactive surface antigen (anti-HBs) antibodies (HBsAb titer ≥10 IU/mL) as a result of immunization or natural infection [18]. Transplantation of organs from an HBsAg+ to naïve unvaccinated patients (HBsAg negative and anti-HBs-negative recipient) is usually not recommended except in the setting of emergency transplantation or in HBV hyperendemic geographical areas. However, transplanting organs from HBsAg+ donors to naïve vaccinated or unvaccinated patients, with human immunoglobulin against HBsAg (HBV-Ig) and antiviral prophylaxis is currently allowed by the Italian guidelines, based on positive preliminary experience [8, 10]. Transplant recipients of HBsAg+ organs should receive HBV-Ig, starting in the intraoperative phase, plus a high barrier nucleos(t)ide analogue (NA) regardless of the immune status, whose duration may significantly vary depending on local center protocols, the transplanted organ (with shorter duration for non-liver recipients), the presence of coinfections (HIV, HDV). Figure 2 summarizes expert opinion recommendations. High barrier NAs have proven to be highly effective, with a successfully suppression of viral replication for the long term with minimal risk for drug resistance, although prophylaxis does not prevent transmission of infection universally. Treatment using tenofovir disoproxil fumarate, tenofovir alafenamide, and entecavir is currently preferred over lamivudine [7]. Laboratory and radiological monitoring after transplantation is recommended to rule out potentially acquired HBV after transplant (Figure 2).

HBsAg+ donors need to be screened to rule out the presence of a Delta virus (HDV) coinfection. Of note that the presence of HBV-DNA in the absence of HBsAg + does not require HDV research. The HDV infection is documented by the positivity of the anti-HDV IgG or IgM. In case of positive anti-HDV-IgG or IgM the presence of an active infection should be ruled out by the determination of plasma HDV-RNA [19].

Liver transplantation from an HBsAg+ and anti-HDV positive donor, according to the Italian guidelines, can be perfumed only in HBsAg+ and anti-HDV positive recipients. On the contrary liver transplantation from an HBsAg+ and anti-HDV negative donor is contraindicated in recipients with HBV-HDV co-infection, because of the risk of HDV infection of the new graft and potential subsequent graft loss [10, 20]. Grafts from donors with isolated anti-HBcAb positivity can be safely used in HBsAg + and anti-HDV positive recipients [7]. Currently there is no approved treatment for HDV after transplant and most effective method for preventing HDV infection of transplanted liver in these patients is dependent on preventing HBV recurrence, with an indefinite combination of NAs with anti-HBV Ig [21]. Interferon remains an option for HDV infection, with poor efficacy and the risk of inducing liver rejection, whereas further studies are needed to determine the role of bulevirtide in the context of liver transplantation (LT) [21, 22] (Figure 1).

**FIGURE 3:**
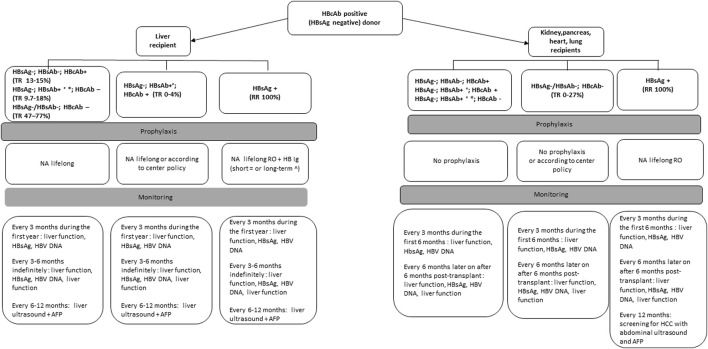
Management of recipients of organs from HBcAb positive, HBsAg negative donors based on expert opinion recommendations.

Isolated anti-HBc positive donors warrant specific consideration, since HBV may persist in the liver with covalently closed circular DNA (cccDNA), which currently cannot be cleared by the host immune response and by antiviral therapies [23]. The risk of transmission from donors with isolated anti-HBc will depend on the immunologic status of the recipient and the type of transplanted organ. Anti-HBc positivity may be seen as 1) false-positive result, especially if risk factors for HBV are absent 2) early hepatitis B infection 3) or resolved infection (HBcAb+, HBsAb-). The risk of transmission of infection from an HBcAb+, HBsAg negative, HBsAb ± donor to a susceptible non-liver recipient is low and recipients with HBV protective immunity do not need antiviral therapy post-transplantation, but careful monitoring and antiviral therapy at the earliest sign of HBV transmission is recommended for recipient management [24]. In contrast for liver recipients, it is recommended to start antiviral prophylaxis and to perform consecutive laboratory testing for HBV infection after transplantation [25] (Figure 3).

No studies have been performed to assess the optimal frequency and type of monitoring for the development of *de novo* hepatitis after transplantation. For recipients of anti-HBc+ livers, most of the studies have described initial monitoring every 1 ± 3 months for 1 year and every 3 ± 6 months after 1 year. For non-liver recipients, optimal monitoring intervals have not been established but we suggest monitoring of serological markers of HBV every 3 months for the first year (Figures 2, 3).

## Hepatitis C Positive Donors

The introduction of direct‐acting antiviral agents (DAA) has produced several consequences in SOT because the number of patients with HCV related cirrhosis and the number of anti-HCV+ viremic recipients in the waiting lists has significantly reduced, the number of HCV+ non viremic individuals in the general population has increased, and allowed the possibility to successfully treat HCV after transplantation [26]. Overall it has also open the option of the use of HCV viremic organs in HCV negative recipients expanding the donor pool without impairing short-term transplant outcomes [27–30].

HCV serological screening should be performed in all donors for the detection of HCV antibodies (anti-HCV) using CLIA techniques or third-generation enzyme immunoassay (EIA), with a sensitivity of at least 95%. HCV RNA screening should be performed to rule out viremia in all anti-HCV+ donors during the donation process and in non standard-risk donors (Table 1). For non standard-risk donors HCV-RNA detection is indicated to reduce the window period from ≈66 to 70 days (antibody detection) to ≈ 5–7 days (eclipse period using NAT) (Figure 1). In the United Sates, screening by NAT for HCV has been mandatory for all organ donors since 2017, regardless of the risk criteria identified during donor evaluation, to reduce the diagnostic window period [31].

The transmission of infection from an anti-HCV+ non-viremic (HCV RNA negative) donor is exceptional (with a low risk for heart, kidney, pancreas and lung and potentially higher risk for liver recipients) but anti-HCV+ viremic donors (HCV RNA positive) transmit HCV infection to almost all recipients, regardless of the transplanted organ [32, 33]. All anti-HCV positive liver donors (both HCV-RNA positive or negative) must be evaluated histologically to exclude the presence of fibrosis [34].

The organs of an anti-HCV+ non-viremic donor (after effective treatment or spontaneous clearance) may be used in anti-HCV positive recipients without restrictions or may be used in anti- HCV negative recipients that accept the risk after informed consent and with close monitoring and treatment in case of transmission [14, 29, 35].

Donation of organs from an anti-HCV+ viremic donor can be performed in HCV viremic recipients or in an anti-HCV negative recipient, if allowed by the national law, who agrees to take the risk after informed consent. In each case early treatment with DAA is strongly recommended [27, 35–38]. Use of liver allografts from HCV-viremic donors to previously treated HCV RNA- negative recipients has also been done with successful DAA retreatment after transplant [39].

It is advisable to determine the viral load and the HCV genotype of the donor, both relevant to recipient management after transplantation. HCV antiviral therapy may be started in the recipient at transplant or as soon as possible early post‐transplant depending on the national rules for DAA reimbursement policies [10, 28, 35, 40, 41]. Standard DAA duration of treatment (12 weeks) is usually recommended but short courses (8 weeks) and ultrashort duration of treatment (≤8 days) may be efficacious in certain settings [35].

Drug interactions between immunosuppressive and DAA should be monitored after transplantation. Recipients of organs from anti-HCV positive donors (HCV-RNA positive or negative) should be monitored by quantitative HCV-RNA determination in peripheral blood at 1, 2, 4, 8 and 12 weeks after transplantation [14].

## HIV Positive Donors

Management of human immunodeficiency virus (HIV) has come a long way since the harrowing days of the 1980s. Currently, life expectancy for a person living with HIV who engages with care shortly after diagnosis now approaches that for the general community. Transplantation is an accepted option for candidates who are themselves living with controlled HIV. It is also expanding as an option for both living and deceased donors [26, 42].

Organ donation from HIV+ patients is available in the United States under the HIV Organ Policy Equity (HOPE) Act, and is now available in multiple other countries, depending on local laws [43]. Much of the early experience came out of South Africa, where using HIV+ kidney donors exclusively for HIV+ recipients has been an option for more than a decade [44]. Almost all global experience subsequently has been in HIV D+/R+ situations, with very rare exceptions (HIV D+/R−) that demand careful legal, ethical and medical caution [45].

For potential deceased donors, organ quality should be examined as per center standards. Patients dying of Acquired Immune Deficiency Syndrome (AIDS)-defining opportunistic infections or cancer are not eligible for organ donation. On the basis of previous literature, in US setting, most (around 60%) of HIV D+ were AntiRetroviral Therapy (ART) experienced which contrasts the South African cohort with the majority (92%) of HIV D+ being ART-naïve. However, even with an ART experienced donor pool, there were no events of HIV breakthrough and no evidence of donor-derived superinfection [44, 46, 47]. Otherwise, assessment should be made regarding the risk of transmitting resistant virus to a recipient both for ART experienced and for ART naïve donors. When a clinician examines a potential donor and notes a clear history of antiretroviral compliance and viral suppression, they should be able to confirm that the antiretroviral treatment of the recipients will also maintain viral suppression of any donor virus. Generally, these are acceptable situations [48]. However, it is the donor with a protracted history of non-compliance or drug resistant virus who needs particular attention, and in some instances, good quality organs should be declined if post-transplant viral control cannot be ensured. Notably many people still do not know of their HIV status, perhaps only being tested when they become a potential organ donor. These individuals might be good donors, not having had an opportunity to acquire more drug resistance, however the possibility of a drug resistant virus should be considered [41, 49].

Centers should be aware that testing for donor evaluation are designed to be particularly sensitive, but consequently can lead to false positives, particularly antibody, or antibody/antigen tests. In recent US HIV transplant cohorts, up to 30% of donors testing positive for HIV were ultimately found to be false positives [50]. HIV-NAT screening is recommended in non-standard risk donors but HIV-NAT positive donors are much less common (Table 1). Donor viral load does not appear to negatively impact organ quality and graft survival, similarly donor CD4 count at terminal illness should be interpreted with caution, as the absolute value may fall significantly during terminal illness, and does not reflect ultimate graft and recipient outcome [51, 52].

For HIV-positive living donors, additional assessments are required, but the small number of outcomes so far have been reassuringly positive for both donors and their recipients [53]. Not only are organ quality characteristics important, but long-term donor renal health must be considered. Historic data would suggest a more rapid decline in living donor residual renal function, although contemporaneous data from an era where integrase inhibitors have dominated care is lacking. A living donor consent conversation should recognize these unknowns [54].

Early data from HIV D+/R+ is promising, however a few caveats are notable. Firstly, rejection rates in the recipients appeared higher when compared against HIV-positive recipients receiving HIV-negative organs. The reasons for this remain unknown. Additionally, in liver recipients, cancer-free survival appeared statistically worse, although numbers were small. These potential detrimental factors should be balanced against an expanded donor pool and shorter transplant waitlist when reviewing potential donor-recipient matches [46, 55].

## COVID-19 Positive Donors

Since the emergence of the Severe Acute Respiratory Syndrome Coronavirus (SARS-CoV-2), there has been a significant impact on organ transplant numbers throughout the world. Especially during 2020, transplant rates fell, as centers tried to prevent spread not only to recipients, but also to healthcare workers. Well documented cases of donor-derived SARS-CoV-2 transmission exist, including transmission to transplant team members [56]. Concern regarding organ quality also led to many potential organs offers being turned down, given the inflammatory nature of Coronavirus Disease-2019 (COVID-19) [57].

Over the course of the pandemic, reassuringly a number of things have changed that allow for the preservation of the donor pool despite ongoing community transmission. Firstly, diagnostics that were so lacking in early 2020 are now widely available, including both point of care testing and molecular testing. Organ procurement policy in many jurisdictions has required testing of potential donors, including the lower respiratory tract if lung donation is considered. Most centers also test transplant candidates, especially those who are symptomatic at the time of organ offer.

Secondly, fear of inflammatory damage to a donated organ has fallen as community levels of immunity have risen. Good quality vaccines are now available across the globe, such that all recipients, and ideally all healthcare workers should be vaccinated. With so many potential donors also vaccinated and/or naturally infected, immunity is such that widespread tissue coagulation and now hyperinflammation are rarely seen. Consequently, even if a donor tests positive for SARS-CoV-2 at the time of donation, clinicians can proceed with confidence that graft organ function is unlikely impaired [58].

Third, treatment options are now widely available and well-studied in the immunosuppressed patient population. Intravenous remdesivir remains a first line treatment agent for acute COVID-19 in any transplant recipient who develops symptomatic disease. Treatment of recipients of non-lung organs is likely unnecessary as now good data suggests these are unlikely to be infectious.

Fourth transplantation of non-lung organs from donors with active SARS-CoV-2 infection is considered possible and well tolerated, without SARS-CoV-2 transmission. There are no documented donor-derived transmission events to liver, kidney or heart recipients [59]. Lung donation from SARSCoV‐2 NAT + donors is generally not recommended, outside two potential approaches [60]. The first is to recover lungs from SARS‐ CoV‐2 NAT positive donors only when symptom onset or test positivity occurred >20 days prior. The second is to recover all organs from asymptomatic SARS‐CoV‐2 NAT positive donors, stratifying the risk of disease transmission using the Ct value. The former emphasizes safety while the latter maximizes organ utilization at the expense of a higher risk of disease transmission given limitation of Ct values to determine infectivity [59, 61, 62]. Finally the use of subgenomic RNA, a proposed surrogate marker of active virus replication, might help to guide organ utilization although this technique is not widely available [63]. There are no reports of using intestinal organ from COVID-19 donors. Given the intestinal tract can be a reservoir for SARS-CoV-2, and intestinal transplant is rarely if ever urgent, this is not routinely advised.

Healthcare worker protection should still be paramount for transplant teams. Generally speaking, any donor who tests positive for SARS-Cov-2 should be managed as potentially infectious. However, once the organ has been procured, this is likely no longer the case, and centers who use positive donors manage infection control at their hospital as per routine. Lung donors with positive SARS-CoV-2 tests should, however, be managed as if they are potentially infectious, as should their recipients after transplant, until suitable tests confirm no transmission [59].

On the basis of the current experience, transplantation of non-lung organs from donors with active SARS-CoV-2 infection has been associated with good short-term outcomes, in terms of 30-day graft loss and mortality. However, studies with longer follow up (6–12-month) found significantly higher rates of hepatic artery thrombosis among recipients of liver and kidney grafts and higher mortality among recipients of hearts obtained from donors with active SARS-CoV-2 [60, 64, 65]. Further studies are needed to assess the long-term outcomes of recipients of organs from donors with active SARS-CoV-2 infection.

## Bacteremic and Candidemic Donors

Blood donor cultures should be obtained routinely at the time of organ donation and prompt transmission of information on blood culture positivity to the recipients’ centers should be done in the shortest time possible and with the highest quality [66, 67]. It has been estimated that 5%–7% of organ donors have bacteremia at the time of organ procurement, but the transmission of the infection to the recipient is low and it has been mainly described in donors with bacteremia due to microorganisms resistant to perioperative antibiotic prophylaxis used in transplantation [68, 69]. In general, liver recipients may be at higher risk of donor transmitted bacteremia compared with recipients of non-hepatic organs and Gram‐ negative bacilli (GNB) appear to pose a greater risk for transmission and are associated with poorer outcomes compared with Gram‐positive bacteria (GPB), except for *S. aureus*, which is a potentially more virulent GPB [70–72].

Transmission of bacterial infections from a donor with bacteremia has been associated with serious consequences for the recipient including overwhelming infection, vascular anastomosis dehiscence in the graft resulting in transplantectomy and death. Additionally, there is controversial information on the relationship between bacteremia in the donor and worsening of graft function [73]. In the same way, there is evidence that demonstrates that the administration of effective antimicrobial therapy in both donor and recipient at the time of the donation process, decreases dramatically (but not eliminates) the risk of transmission, making this practice reasonably safe.

In general organs from donors with positive blood cultures may be safely used if they have received an appropriate antimicrobial for at least 24–48 h, ideally with some degree of clinical response (improved white blood cell count, improved hemodynamics, defervescence of fever), since in clinical practice documented clearance of donor bacteremia is often not achievable before transplantation.

In addition, a complete course of therapy (range 7–14 days) depending on the presence of virulent microorganism (such as *S. aureus* and *P. aeruginosa* in particular) should be given to the recipient post-transplant with targeted antimicrobial treatment. Donors with documented bacteremia should be used with informed consent, after evaluation of the transplant infectious diseases team, and recipients should undergo systematic surveillance cultures after transplantation.

Endocarditis does not constitute a contraindication for transplantation, except for heart. The use of organs from donors with infective endocarditis remains controversial for the risk of metastatic infections but can still be used based on individual decision [74]. Ideally patients with endocarditis can be accepted as donors of non-heart organs if they have received proper antibiotic treatment prior to donation (preferably a minimum of 24–48 h), if they have cleared blood cultures and there is no evidence of peripheric emboli that have damaged the organs to be transplanted. The recipient must continue treatment for at least 10–14 days with active drugs, whose choice and duration must be modulated according to the results of the blood cultures of the donor at the time of organs procurement.

Non-bacteremic localized infections from other sites only require antibiotic treatment if transmission in the transplanted organ is plausible (positive urine cultures for kidney recipients; respiratory cultures for lung recipients) but it is not recommended for the other organs recipients. The donor with localized bacterial infection must have received adequate treatment prior to donation (preferably a minimum of 24–48 h). Targeted antibiotic treatment should be continued in the recipient of the infected organ.

Most cases of donor-derived candidiasis have occurred in kidney transplant recipients and rarely in liver transplant recipients in whom contaminated preservation fluid is a commonly proposed source, but also donor candidemia without effective antifungal therapy can be infection source [75, 76]. In this setting DDI fungal infection can result in life-threatening complications like arteritis and vascular aneurysms. On the basis of our national protocol, transplant of donors with untreated candidemia is not recommended and donors with positive blood cultures for *Candida* spp. can be accepted only after 24–48 h of effective antifungal therapy prior to organ procurement and recipients should receive at least a 14‐day course of antifungals (echinocandins are the preferred antifungal therapy) targeting the donor *Candida* spp. isolate [10].

## Multidrug Resistant Bacteria and Fungi

Transmission of most bacterial infections may be prevented by the use of surgical prophylaxis at time of transplant surgery, but due to the emergence of multidrug resistant (MDR) bacteria, routine prophylaxis might fail to prevent transmission of bacteria from the donor organ at the time of procurement [77, 78]. Gram-positive MDR bacteria (vancomycin-resistant *Enterococcus* species, methicillin-resistant *Staphylococcus aureus*) do not appear to have a significant impact on organ utilization [79]. On the contrary MDR Gram-negative bacteria (MDR GNB), which include, carbapenem-resistant *Pseudomonas aeruginosa*, carbapenem-resistant *Acinetobacter baumannii*, *Klebsiella pneumoniae* and other carbapenem-resistant Enterobacterales, has been observed to reduce organ procurement and transplantation [79, 80]. There is no evidence to suggest that organs from donors infected or colonized with Extended-spectrum β- lactamase—producing Enterobacterales (ESBL) be excluded from transplantation [81, 82].

Transmission with organ transplantation of MDR-GNB organisms has been associated with serious consequences for the recipients in terms of morbidity and mortality [71, 83, 84]. There is limited experience on risk mitigation strategies related to MDR-GNB bacteria that have been successfully implemented to minimize the impact of MDR-GNB donor‐transmitted bacteria following organ transplantation. Indeed, limited reports showed that recipients of organs from donors with MDR-GNB infection may have a favorable outcome with early microbiological diagnosis, peri-transplant targeted antibiotic therapy due to successful intra- and inter-institutional communication and prolonged treatment after transplantation [67, 85–87]. These results underline that active surveillance system should be implemented to avoid communication gaps that might be associated with infection transmission and could allow the policies on the use of organs from MDR-GNB positive donors to be reconsidered [87]. Rapid and effective interagency and interinstitutional communication regarding donor cultures are imperative to optimize recipient management [81].

In addition, the current availability of new drugs with activity against some MDR-GNB pathogens and new possible decontamination techniques performed after organ procurement might allow in the future a more liberal use of these organs [85, 88]. However further work is needed to understand how to prospectively identify donors that may harbor MDR subclinical infection, and how to best manage recipients at risk for MDR-GNB donor‐derived infections following transplantation [89].

In general, the confirmed presence of MDR-GNB bacteremia constitute an exclusion criterion from the donation, because outcomes in such circumstances are still unknown, but individual donor evaluation is required with careful discussion with the transplant infectious diseases team. The efficacy of appropriate antimicrobial treatment of the donor before organ procurement on the basis of *in vitro* susceptibility data, in preventing recipient infection, is not known. Risk‐benefit assessment is needed to drive decisions to accept the organ but a clear plan for effective peri- and post-transplant antibiotics for the recipient should be outlined prior to the use of such organs [78, 89]. As regards as localized infections (pneumonia, infections of the urinary tract), in the absence of associated bacteremia, the exclusion applies only to the infected organ [90].

There is insufficient data to determine the risk of transmission of infection from a donor colonized by MDR-GNB to a recipient. The isolated positivity of the rectal swab for MDR-GNB should not be considered a criterion of exclusion from donation, except for bowel and pancreas donation and requests the highest respect for surgical aseptic procedures in order to avoid contamination of the procured organs [78, 90].

It seems prudent to exclude organs colonized or infected by MDR GNB (lungs, kidney) although in specific situations the organs colonized with MDR bacteria may be safely used when the recipients receive prompt tailored antibiotic treatment [91]. It is not currently recommended administration of modified antibiotic prophylaxis to recipients of organs from donors that are colonized but it is important to have the microbiological donor history recorded in order to adjust the empirical antibiotic treatment in case of suspected infection immediately after the transplantation [78, 90].

*Candida auris* is an emerging pathogen capable of drug resistance and persistence in the environment with important public health implications and has several implications for organ transplantation. The possibility of donor-derived transmission of *C. auris* has been described [92]. Isolation from an organ donor warrants careful consideration before transplantation. At present, there are few data to guide such decisions.

## Conclusion

Donor-derived infections continue to be a challenge. Awareness of epidemiological changes and emerging pathogens alongside the improvement of rapid and reliable microbiological screening are basic tools to improve organ safety and quality of organs allocation. It is vital to develop prospective and high-quality research to improve a more tailored approach and knowledge on short- and long-term outcomes of DDIs. Moreveor new frontiers need to be explored to expand the donor pool demanding careful legal, ethical and medical caution.

## Abbreviations

AFP, alpha fetoprotein; COVID-19, Coronavirus Disease-2019; D, donor; DAA, direct‐acting antiviral agents; DO, donor oriented; HCC ENT, entecavir, Hepatocellular carcinoma; LAM, lamivudine; MDR, multidrug resistant; NA, nucleos(t)ide analogue; NAT, nucleic acid testing; R, recipient; RO, recipient oriented; RR, reactivation risk; SARS-CoV-2, Severe Acute Respiratory Syndrome Coronavirus; SOT, solid organ transplant; TDF, tenofovir; TR, trasmissione risk (without propylaxis).
